# Epigenetic-Based Therapy—A Prospective Chance for Medulloblastoma Patients’ Recovery

**DOI:** 10.3390/ijms22094925

**Published:** 2021-05-06

**Authors:** Agata Strejczek, Dawid Woszczyk, Helena Urbaniak, Martyna Różańska, Michał Robak, Zofia Matuszewska, Anna-Maria Barciszewska

**Affiliations:** 1 Medical Faculty, Karol Marcinkowski University of Medical Sciences, Fredry 10, 61-701 Poznan, Poland; strejczek.agata@gmail.com (A.S.); d.woszczyk99@gmail.com (D.W.); helur@onet.pl (H.U.); martyna.ro899@gmail.com (M.R.); mrobak101r@gmail.com (M.R.); zofia.matuszewska99@gmail.com (Z.M.); 2 Intraoperative Imaging Unit, Chair and Clinic of Neurosurgery and Neurotraumatology, Karol Marcikowski University of Medical Sciences, Przybyszewskiego 49, 60-355 Poznan, Poland; 3 Department of Neurosurgery and Neurotraumatology, Heliodor Swiecicki Clinical Hospital, Przybyszewskiego 49, 60-355 Poznan, Poland

**Keywords:** medulloblastoma, epigenetics, targeted therapy, DNA methylation, DNA methyltransferase inhibitors, histone deacetylases inhibitors, miRNA, lncRNA, bromodomain

## Abstract

Medulloblastoma (MB) is one of the most frequent and malignant brain tumors in children. The prognosis depends on the advancement of the disease and the patient’s age. Current therapies, which include surgery, chemotherapy, and irradiation, despite being quite effective, cause significant side effects that influence the central nervous system’s function and cause neurocognitive deficits. Therefore, they substantially lower the quality of life, which is especially severe in a developing organism. Thus, there is a need for new therapies that are less toxic and even more effective. Recently, knowledge about the epigenetic mechanisms that are responsible for medulloblastoma development has increased. Epigenetics is a phenomenon that influences gene expression but can be easily modified by external factors. The best known epigenetic mechanisms are histone modifications, DNA methylation, or noncoding RNAs actions. Epigenetic mechanisms comprehensively explain the complex phenomena of carcinogenesis. At the same time, they seem to be a potential key to treating medulloblastoma with fewer complications than past therapies. This review presents the currently known epigenetic mechanisms that are involved in medulloblastoma pathogenesis and the potential therapies that use epigenetic traits to cure medulloblastoma while maintaining a good quality of life and ensuring a higher median overall survival rate.

## 1. Introduction

Medulloblastoma is one of the most common tumors of childhood and accounts for 20% of all pediatric brain tumors [1], with a peak incidence between 3–9 years of age [2]. MB is extremely rare in adulthood and accounts for less than 1% of intracranial tumors [3]. Medulloblastoma is more common in males than females [4], but there is no correlation with race [5]. The typical location of the tumor is the posterior fossa, and therefore, it has a high tendency to disseminate via cerebrospinal fluid pathways. Spread beyond the central nervous system is very rare [6]. Most medulloblastomas develop within the cerebellar vermis, causing increased intracranial pressure through a tumor mass and hydrocephalus, as well as cerebellar dysfunction [7]. The current treatment, including surgery, craniospinal irradiation, and high-dose chemotherapy, leads to improved survival rates despite the high malignancy of MB [7]. The currently achieved rates are 82 ± 5.2% and 76 ± 5.8% for median overall survival and 2-year progression-free survival, respectively [8]. Nevertheless, these aggressive treatments carry a high risk of complications and neurological deficits [9,10]. Therefore, the low quality of life connected with neurocognitive, neurosensory, and endocrine deficits, as well as a high risk of tumor recurrence, force the need to develop new, tumor-specific therapies that spare healthy brain tissue [7].

Because medulloblastoma is a heterogeneous disease, the prognosis and cure rate strongly depend on the cellular abnormalities [11]. One can distinguish histological variants of MB with clinical utility: classic, desmoplastic/nodular (DNMB), extensive nodular (MBEN), or large cell/anaplastic (LC/A) [12]. The classic type of MB is more common in childhood, while DNMB is more common in infants and adults [13]. Medulloblastoma with extensive nodularity in infants has a good prognosis, as does the DNMB type at young ages [12]. However, histological subtyping has several limitations. It omits a vast amount of information about the tumor biology at a molecular level [14]. Therefore, a 2016 WHO revision introduced a new medulloblastoma classification based on defined molecular characteristics. It distinguishes four subtypes: WNT (wingless), SHH (sonic hedgehog), group 3, and group 4. Each subtype shows its pattern of gene expression, clinical presentation, and location [12,15,16]. SHH is most frequent among infants and adults, while group 3 dominates in children [17]. Transcription factor p53 mutations are frequent in the WNT (16%) and SHH (21%) subgroups, but in the WNT subgroup, p53 mutation has no prognostic implication (5 y OS in 90% and 97% for p53 mutant and WT, respectively) [18]. In SHH, the presence or absence of a p53 mutation changes the prognosis significantly (5 y OS in 41% and 81% for p53 mutant and WT, respectively) [18] (Table 1).

## 2. Epigenetic Changes in Medulloblastoma

The molecular subgrouping and biology of MB, in conjunction with histopathology, are increasingly driving prognostication. However, many aspects of MB carcinogenesis, such as the mechanisms underlying metastatic disease, remain poorly understood [24]. Therefore the next source for providing more details on tumor pathogenesis, and an opportunity for personalized treatment, is epigenetics.

Epigenetics is the sum of heritable alterations in gene expression occurring without changes in the DNA sequence. It provides a novel explanatory area for the physiological and pathological processes in the cell. It offers a mechanistic bridge between genetic and environmental factors that influence the development of the disease. Epigenetic regulation of gene expression is a dynamic, responsive, and reversible process that establishes a normal cell. It has also been shown to contribute to human disease progression and severity [25]. Many types of epigenetic processes have been identified, which include DNA methylation, histone modifications, and noncoding RNAs. Epigenetic processes are natural and essential to all organism functions, but their disturbance can have major adverse health and behavioral effects.

### 2.1. Histone Modifications

Chromatin modification is an important epigenetic process that regulates the accessibility of DNA to regulatory proteins, such as transcription factors and DNA binding proteins. This can involve histone methylation (of lysine or arginine), as well as acetylation (of lysine) [26]. Specific methyltransferases and demethylases [27,28], as well as acetyltransferases and deacetylases [29], control these processes. Histone methylation is mainly responsible for controlling transcription via its activation or repression [30]. Lysine methylation also works with DNA methylation, which helps to regulate gene expression [31]. Disturbances in histone methylation, for example, a mutation in the methyltransferases’ genes, lead to the development of diseases, including neoplasms [32]. In the case of medulloblastoma, one can observe an overexpression of histone demethylase KDM1A, which causes the demethylation of lysine 4 in histone 3 (H3K4), which is connected with a poor prognosis [33]. Histone acetylation stabilizes euchromatin and activates transcription. This is possible thanks to specific acetyltransferases, which add an acetyl group to histone’s lysine. In this reaction, its positive charge becomes neutral, which allows for chromatin relaxation [34]. Simultaneously, deacetylation condenses chromatin to heterochromatin, leading to transcription repression due to the deacetylation histones gaining positive charge and binding to the negatively charged DNA [35]. This process is carried out by histone deacetylases (HDACs). HDACs were also found to deacetylate other proteins participating in the gene expression, such as α-tubulin, DNA repair enzymes, and transcription factors themselves [35]. They can be divided into several groups, including classes I (HDACs 1, 2, 3, 8), II (HDACs 4, 5, 6, 7, 9, 10), and IV (HDACs 11), which are the targets for HDAC inhibitors [36]. One can observe the role of acetylation and deacetylation in MB development. Lysine acetyltransferase 2B (PCAF) is an important cofactor of the Hedgehog-GLI (Hh-GLI) signaling pathway, which is responsible for medulloblastoma pathogenesis [37]. Histone deacetylation silences Dickkopf WNT Signaling Pathway Inhibitor 1 (*DKK1*), which is an antagonist of the WNT signaling pathway and is important for medulloblastoma development [38].

### 2.2. MicroRNAs (miRNAs, miRs)

MicroRNAs are small noncoding RNA molecules (containing about 22 nucleotides) that function in the RNA silencing and post-transcriptional regulation of gene expression [39,40]. It is assumed that 50% of miRNAs are transcribed from non-protein-coding transcripts, and the remaining ones are encoded in the introns [41]. MicroRNAs regulate gene expression by affecting the mRNA, mainly through pairing with their imperfect complementary sites within the 3′-untranslated regions (3′UTRs), but also the 5′UTRs [42] and coding sequences [43]. miRNAs influence WNT, SHH, and mitogen-activated protein kinase (MAPK) pathways as transcriptional repressors [44]. Therefore, the dysregulation of miRNAs’ activity is tumorigenic; for example, miR-326 downregulation promotes the SHH signaling pathway, which is responsible for medulloblastoma development [45]. Another example is the miR-17/92 cluster overexpression that is related to activated SHH/PTCH pathways and influences medulloblastoma growth [46,47]. In MB pathogenesis, one can observe a lot of miRNAs’ expression dysregulations [48] (Table 2).

### 2.3. Long Noncoding RNAs (lncRNAs)

lncRNAs are defined as non-protein-coding transcripts that are longer than 200 nucleotides. They regulate embryonic pluripotency, differentiation, and patterning of the body axis, and promote developmental transitions. Moreover, lncRNAs function as molecular scaffolds regulating histone modifications and influence the epigenetic programs of the transcriptome [62,63].

In medulloblastoma, one can observe dysregulations of lncRNA. Some of them affect miRNA activity (miRNA sponging) and pathways of medulloblastoma development (MAPK, SHH, PI3K/AKT). These dysregulations usually lead to the promotion of cell proliferation and migration [64]. Table 3 presents the most known lncRNAs that affect medulloblastoma development. Recent findings suggest that lncRNAs could potentially be prognostic and diagnostic markers in medulloblastoma [65].

One example of an lncRNA, namely, colorectal neoplasia differentially expressed (*CRNDE*), has been shown to promote medulloblastoma growth [66] and induce drug resistance [67]. The medulloblastoma cell lines, Daoy and D341, which have a high level of *CRNDE* transcript, also have significantly higher LC_50_ values after exposure to cisplatin in comparison to D283 and D425 cell lines, which have a lower level of *CRNDE* transcript [67]. The potential mechanism of this phenomenon is miR-29c-3p overexpression in Daoy and D341 cells, which results in the suppression of medulloblastoma cells’ proliferation and migration. Furthermore, it induces tumor chemosensitivity to cisplatin. However, an excess of *CRNDE* acts in the opposite way: it sponges the miR-29c-3p and, therefore, depletes its function. Consequently, this type of medulloblastoma develops drug resistance [67]. This defines *CRNDE* as a potential target in medulloblastoma treatment [67].

**Table 3:** Dysregulated lncRNAs in MB and their functional targets.

lncRNA	Expression [Up- (↑) or Down- (↓) Regulation]	Target		Results [Increases (↑) or Decreases (↓)]	MB Subgroup	Ref.
		Direct (Sponging Activity)	Indirect			
LOXL1-AS1	↑	-	PIK3–AKT pathway	↑ cell proliferation and metastasis	-	[68]
TP73-AS1	↑	miR-494-3p	*EIF5A2*	↑ cell proliferation, migration, invasion, and colony formation; ↓ apoptosis	-	[69,70]
HOTAIR	↑	miR-1 and miR-206	*YY1*	↑ tumor growth, migration, invasion, and epithelial-to- mesenchymal transmission; ↓ apoptosis	-	[71]
CCAT1	↑	-	*MAPK, ERK, MEK*	↑ cell proliferation and metastasis	-	[71]
CRNDE	↑	miR-29c-3p	-	↑ tumor growth; ↓ chemosensitivity	-	[72,73]
UCA1	↑	-	-	↑ cell proliferation, migration, invasion, metastasis, and angiogenesis	-	[74,75]
SPRY4-IT1	↑	-	*MMP-2*	↑ cell proliferation, migration, and invasion	-	[76]
ANRIL	↑	miR-323	*p38, MAPK, ERK, AKT, BRI3, CDK6*	↑ cell proliferation and migration; ↓ apoptosis	-	[77]
Linc-NeD125	↑	miR-19a-3p, miR-19b-3p, miR-106a-5p	*CDK6, MYCN, SNCAIP, KDM6A*	↑ cell proliferation, migration, and invasion	Group 4	[78]
lnc-HLX-2-7	↑	-	*HLX*	↑ cell proliferation, viability, and colony formation; ↓ apoptosis	Group 3	[79]
Nkx2-2as	↓	miR-103/107, miR-548m	SHH pathway	tumor development	-	[80]

### 2.4. Methylation

DNA methylation, which is a dynamic process involving methylation and demethylation events, occurs in different regions of the genome and is crucially important for embryogenesis, cellular proliferation, and differentiation [81,82]. This process is catalyzed by DNA methyltransferases (DNMTs), which add a methyl group to cytosine, forming 5-methylcytosine (m^5^C), the main epigenetic mark. In general, methylation occurs when cytosine precedes guanine in a DNA chain (a place known as a CpG site). The methylation pattern of each cell, tissue, and organism is specific. There are several types of DNMTs. DNMT1 works along with DNA replication to save the methylation pattern. DNMT3a and DNMT3b are de novo methyltransferases [83]. No enzymes that specifically demethylate DNA have been found so far. However, it has been shown that the removal of m^5^C occurs through the oxidation of m^5^C in the presence of nonspecific TET proteins, which require Fe(II) and ketoglutarate as cofactors [84,85]. In tumor cells, the hypermethylation of suppressor genes and global hypomethylation is observed [86,87]. DNA methylation profile changes are induced by environmental and endogenous factors [88]. In medulloblastoma, one can observe that the activation of the SHH signaling pathway is connected with DNMT1 overexpression [89]. Hypermethylation of antioncogene promotors, such as Hypermethylated-in-Cancer 1 (*HIC1*), ras association domain family 1 isoform A (*RASSF1a*), and caspase 8 (*CASP8*), occurs in medulloblastoma. Silencing them causes tumor proliferation [90,91] (Table 4).

## 3. Epigenetic Therapeutics in Medulloblastoma

Epigenetic machinery regulates gene expression and has a crucial impact on carcinogenesis. Changes in epigenetic networks are more frequent and faster than genetic networks. Therefore, they describe the pathomechanism of carcinogenesis more accurately and comprehensively [92]. Therefore, we looked for the possible application of known and potential epigenetic targets and drugs for treating medulloblastoma.

### 3.1. DNA Methyltransferase Inhibitors (DNMTis)

**Figure 1:**
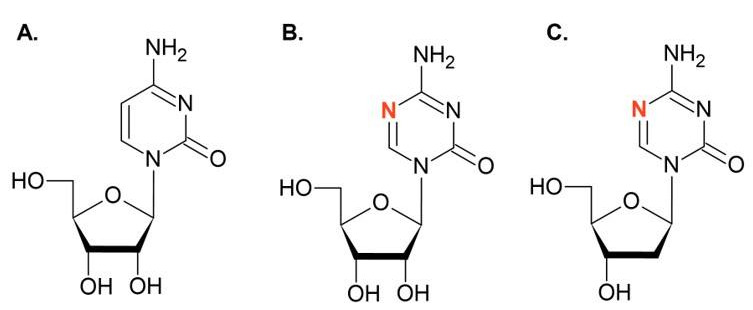
(**A**) Cytidine and its analogs (**B**) 5AC and (**C**) DAC. An additional nitrogen atom (marked in red) is responsible for the inhibitory activity of these molecules toward DNMTs.

Molecules that affect the DNA methylation process, such as DNA methyltransferase inhibitors, have been shown to impact cancer cells [93]. Some of the DNMTis in clinical use are 5-azacytidine (5AC) and 5-aza-2′-deoxycytidine (DAC) [94]. These are chemical analogs of cytidine, where the fifth carbon atom is replaced by a nitrogen atom (Figure 1).

**Figure 2:**
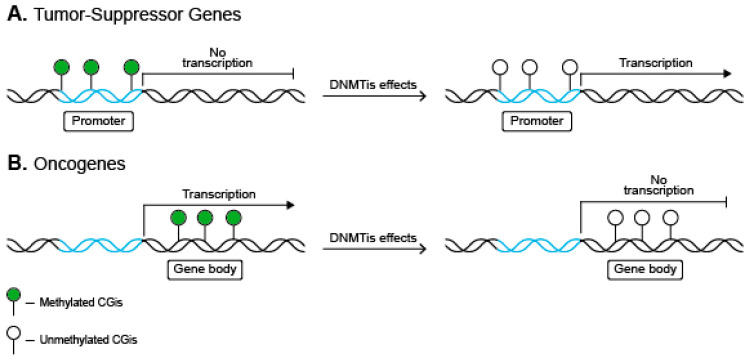
Major DNMTis effects on MB cells. (**A**) The transcription of TSGs is induced by DNMTi-dependant hypomethylation of the gene’s promoter. Encoded proteins regulate cell proliferation. (**B**) In contrast, DNMTi-induced hypomethylation of the gene’s body stops the translation process. In the case of oncogenes, which encode proteins that induce cell proliferation, such hypomethylation induces apoptosis.

DNMTs cannot properly interfere with the nitrogen atom and, therefore, are no longer capable of onward methylation [95]. This results in hypomethylation, which induces anti-oncogene (tumor suppressor genes, *TSG*) expression when occurring in the gene promoter. On the other hand, the hypomethylation of a gene body represses oncogene expression [96] (Figure 2).

Hypermethylation of the *TSG* promoter is a common epigenetic aberration that prevents cell apoptosis and has been identified in MB cells. *HIC1*, *RASSF1a*, and *CASP8* are the *TSG*s in which the promoter is most frequently hypermethylated in MB [90]. DNMTis, used alone or in combinations with other drugs, are used to reactivate those genes. It was shown that DAC significantly decreases the metabolic activity of MB cell lines, with ≈20% of the initial activity remaining [97]. DAC affects proliferating cells. This suggests that the demethylating effects depend on the cells’ capability regarding DNA repair, DNMT activity, and other mechanisms.

Zebularine (Zeb) is another nucleoside-based DNMTi that induces antiproliferative effects in numerous cancer cells [98,99]. It is less potent than 5AC, but unlike 5AC, Zeb is more bioavailable and less cytotoxic [100]. Therefore, the effective doses of the drug are respectively higher. Zeb also exerts anticancer effects in MB cells [101]. It alters the general gene expression, including the SHH pathway members. The decreased GLI1 level in MB cells after Zeb treatment suggests a possible treatment method for SHH MB. Furthermore, p53 and p21 protein levels are higher after being exposed to Zeb. Other genes, whose expression is induced under Zeb, are *BATF2* and *BAX*. Zeb was also studied in combination with vincristine (VCR), showing synergistic effects, but antagonistic effects were observed when combined with cisplatin [101].

**Figure 3:**
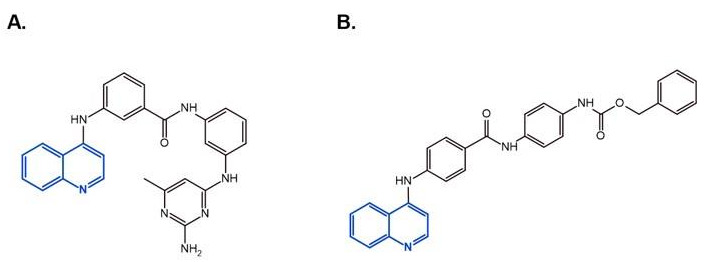
The chemical structures of quinoline-based DNMTis. (**A**) MC3343: one of the first non-nucleoside DNMTis tested in a cancer stem cell line. (**B**) MC3353: a novel, more potent compound.

There are also DNMTis that are not nucleoside-based. Some of them are natural products (e.g., curcumin, epigallocatechin gallate). They bind to the catalytic region of DNMTs and do not interfere with DNA, which results in lower cytotoxicity [36]. However, most of them are less effective and less selective for DNMTs than nucleoside-based DNMTis [36]. On the other hand, compounds called quinoline-based DNMTis are more selective and potent than most non-nucleoside-based DNMTis. They have antiproliferative effects and induce cytodifferentiation in MB cells, which can be measured by quantifying the proliferating cell nuclear antigen (PCNA) levels and MTT assays [102]. MC3343 is an example of an effective non-nucleoside-based DNMTi that was tested on MB cells (Figure 3). Recent findings show that MC3353 is a potential drug that has stronger demethylating activity than both 5AC and DAC. However, its effects on MB cells have not been studied [103].

### 3.2. Inhibition of miRNA

Several miRs have already been explored as therapeutic targets in MB. miR-217 is almost 12-fold overexpressed in MB and is a potential oncogene that negatively regulates *SIRT1*, *SMAD7*, *ROBO1*, and *FOXO3* genes [104]. It has been shown that anti-miR-217 inhibits invasion, proliferation, and migration, and causes apoptosis. Therefore, it could be used in the therapy of group 3 MB, where it leads to the decrease of colony formation in the HDMB03 cell lines [104].

miR-584-5p was identified as a potent suppressor of MB in mice because it affects the effectiveness of VCR and ionizing radiation (IR), which is the basic treatment against MB [59]. It targets eukaryotic translation initiation factor 4e family member 3 (eIF4E3) and histone deacetylase 1 (HDAC1), affecting the cell cycle progression, DNA impaired reaction, and microtubule dynamics. It makes MB cells more sensitive to VCR and IR. The toxic side effects caused by this treatment could be minimized by miR-584-5p thanks to the lower dose of VCR and IR [59].

miR-34a downregulates melanoma-associated antigen A (MAGE-A), causing MB cells to be more sensitive to chemotherapeutics, such as mitomycin and cisplatin [60]. Adenovirus with miR-34a inhibits tumor proliferation in vivo without toxic damage [105].

miR-193a and miR-224 inhibit the proliferation and growth of MB cells, and similar to miR-584-5p, make MB cells more sensitive to the IR [52].

miR-128a decreases MB proliferation, probably via the downregulation of BMI1 protein [61].

miR-199b has a similar effect to miR-34a in vivo and could be applied in high-risk patients. It affects Notch signaling in cancer stem cells by controlling HES1 and suppresses the tumor [51]. In MB with CDK6 overexpression, the potential therapeutic agent is miR-124, whose deficiency can be a reason for this disorder [106]. Intravenous injection of a locked nucleic acid (LNA) inhibits miR-17/92 and decreases tumor growth in SHH MB mice [46].

### 3.3. Histone Deacetylase Inhibitors (HDACis)

Histone deacetylase inhibitors are a heterogeneous group of epigenetic modulators targeting classes I, II, and IV of histone deacetylases [36]. Their other biologic effects include immunomodulatory activity and killing both proliferating and nonproliferating tumor cells [107]. Since different HDACs have distinct impacts on cell function, many isoform-specific or selective HDACis are currently being developed and are undergoing clinical trials [108]. Selected examples of the currently most researched HDACis in medulloblastoma are presented in Table 5.

#### 3.3.1. Suberoylanilide Hydroxamic Acid

Suberoylanilide hydroxamic acid is an inhibitor of HDAC classes I and II. It shows a selective effect on gene expression and induces the activation of the *p21^WAF1^* gene and associated promoter proteins via the acetylation of H3K9,14 and H4K5,8,12. These changes can lead to the differentiation and apoptosis of the medulloblastoma cells [109,110]. Moreover, SAHA has shown synergistic effects in combination with chemotherapeutics, such as Doxorubicin, Etoposide, and Cisplatin, by triggering Bax activation, resulting in caspase-dependent apoptosis [112]. Another SAHA activity is the modulation of the proteasomal degradation of the transcriptional repressor REST (RE1-Silencing Transcription factor). High levels of REST are a poor diagnostic indicator for medulloblastoma patients, while SAHA causes its decline and REST-dependent repression of cell growth [111].

#### 3.3.2. Panobinostat

Panobinostat, a pan-HDACi, shows potent selective inhibitory effects on murine and human MB cells in doses that do not affect the granule neurons or astrocytes. Its greatest potency is against group 3 MB, one of the most aggressive forms of MB, which is characterized by the amplification or overexpression of the *MYC* oncogenes (Table 1). Treatment with Panobinostat causes an increase of the forkhead box protein O1 (FOXO1) target genes, which results in a decreased viability of tumor cells and prolonged survival in mouse models [119]. The specific mechanism of FOXO1 action includes promoting the expression of cyclin-dependent kinase (CDK) inhibitors and proapoptotic proteins (thus leading to cell cycle arrest and apoptosis) and inhibiting the expression of *MYC* target genes in favor of *MYC* antagonists [120]. FOXO1 activity is also regulated by the phosphoinositide 3-kinase (PI3K) inhibitors, which can be used together with HDACis, such as Panobinostat, with a synergistic outcome, namely, inhibition of the growth of *MYC*-driven MB cells [119]. Panobinostat has also been found to reduce the tumor volume and tumor cell proliferation in SHH MB mouse models with *CREBBP* (CREB-binding protein gene) mutations. The CREB-binding protein (CBP) is a transcription co-activator. The therapeutic effect of HDACi in this case probably involves canceling the downregulation of brain-derived neurotrophic factor (*BDNF*) expression, which contributes to the histone acetyltransferase activity of CBP. However, a positive response was not observed with *CREBBP* wild-type SHH MB [121]. In addition, Panobinostat is responsible for the down-regulation of DNA-binding protein inhibitor (ID3), which is a compound that is responsible for spinal leptomeningeal seeding. This translates into a better survival rate [122].

#### 3.3.3. Trichostatin A

Trichostatin A is a class I and II HDACi that is designed to decrease the viability of MB cells and induce their apoptosis by restoring *DKK1* expression. *DKK1* is a WNT antagonist with a considerable tumor-suppressing activity under transcriptional silencing in MB cells. TSA causes the upregulation of *DKK1* via histone acetylation in its promoter region, thus allowing it to inhibit the clonogenic growth of MB [38]. Moreover, TSA-induced inhibition of class I HDACs impairs the SHH-pathway via the acetylation of its key transcriptional factors, namely, *Gli1* and *Gli2*. The synergistic use of the Cul3–REN E3 ubiquitin ligase complex can enhance the suppression of tumor growth [123,124]. Similar to Panobinostat, TSA was also found to be a promising therapeutic option for the treatment of the *CREBBP*-mutated SHH MB [121].

#### 3.3.4. Valproic Acid

Valproic acid is a short-chain fatty acid that inhibits class I and II HDACs, causing hyperacetylation of histone H3 and H4 [125]. Its antitumor activity manifests via the activation of *p21* and the suppression of *TP53*, *CDK4*, and *c-MYC* expression [126]. A recent study reported that VPA may have varying efficacy based on the genetic background of MB cells. In *TP53^WT^* MB cells (from SHH and groups 3 and 4), VPA led to cell cycle arrest and apoptosis, whereas in *TP53^MUT^* MB cells (from the SHH subgroup), it induced resistance to the treatment [127].

#### 3.3.5. Sodium Butyrate

Sodium butyrate, a class I and IIa HDACi, was found to reduce the cell viability and modulate the stemness phenotype of MB by downregulating the expression of stemness markers, such as *CD133* and *BMI1* and ERK activity. CD133-positive cells are invasive in vivo and have higher migration and invasion capabilities than the parental cell line [113]. BMI1 is a polycomb group protein and another poor outcome marker involved in the invasion of MB [114]. This antiproliferative effect of NaB can be reinforced in combination with a MAPK/ERK inhibition, as this pathway is the most enriched process in MB [115].

#### 3.3.6. Quisinostat

Quisinostat is a pan-HDACi with a notable efficacy toward HDAC I [116]. HDAC I is considered the most upregulated group of HDACs in SHH MB, and, together with HDAC II, play a crucial role in the growth of the MB cells. Reducing the expression of only one of them is not sufficient because of the compensatory increase of the other one and overlapping functions. Quasinostat allows for the simultaneous inhibition of HDACs I and II and, thus, abrogation of SHH signaling via downregulating the expression of the SHH target genes, especially the main SHH effector *Gli1* [117]. Moreover, there are many cases of SHH MB, which carry gene mutations that activate the SHH downstream of the Smoothened (SMO) receptor, making them resistant to the common treatment, namely, SMO inhibitors [128]. Since Quisinostat acts downstream to these SHH pathway components, it has been found to inhibit the proliferation of SMO-sensitive, as well as SMO-resistant, SHH MB cells. Mouse models show promising results, including the capability of Quisinostat to cross the blood–brain barrier while also being well tolerated in the organism [117]. Another study demonstrated that Quisinostat induced Daoy and D283 cell apoptosis and G2/M cell cycle arrest via the induction of caspase-3 and poly (ADP-ribose) polymerase (PARP) cleavage and the acetylation of H3 and H4 histones. Daoy and D283 cell lines represent different MB subtypes, making Quisinostat a potential therapeutic agent for a broad MB spectrum [118].

#### 3.3.7. Entinostat

**Figure 4:**
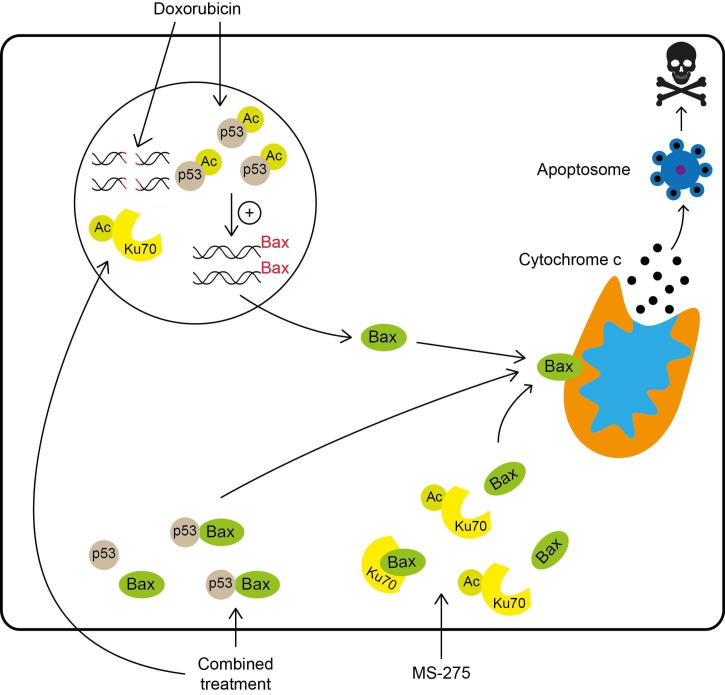
Comparison between treatments with MS-275, Doxorubicin, and their combination, leading to the apoptosis of the MB cells. Monotherapy with Doxorubicin causes the acetylation and nuclear accumulation of p53, which induces Bax expression. Bax protein then oligomerizes to the mitochondrial outer membrane, causing cytochrome c release, which induces apoptosis. Acetylation of the Ku70 protein with MS-275 releases Bax and leads to apoptosis. Combined treatment with Doxorubicin and MS-275 shows a synergistic apoptotic effect. It promotes the binding of p53 to Bax, leading to Bax activation and the loss of mitochondrial membrane potential, as well as greater DNA damage. Ku70, acetylated by MS-275, has reduced DNA repair activity. Thus, DNA strand breaks caused by the Doxorubicin are left unrepaired.

Entinostat is a selective inhibitor of the class I HDACs. Treatment of MB cells with MS-275 causes the acetylation of histone H3 and the non-histone protein Ku70, which leads to the release of Bax from Ku70. Similar to SAHA, MS-275 works together with DNA-damaging drugs, such as Doxorubicin, Etoposide, and Cisplatin, to enhance p53-dependent mitochondrial apoptosis. This combined treatment promotes the binding of p53 to Bax, p53-dependent Bax activation, and results in caspase-dependent apoptosis (Figure 4). The MB growth-suppressing activity of MS-275 and Doxorubicin was also confirmed in an in vivo model [112]. Moreover, an important role of MS-275 in the treatment of group 3 MB (which has an overall poor survival rate) was suggested. As mentioned before, a distinguishing characteristic of group 3 MB is *MYC* amplification. Since the transcriptional factor *c-MYC* controls the transcription of class I HDAC2, it is especially strongly overexpressed in this subgroup [129]. These factors together make group 3 MB cells significantly more sensitive to class I HDACis, such as MS-275, which causes acetylation of the histone H4, reduced metabolic activity, and ends with apoptosis [130].

### 3.4. Bromodomain and Extra-Terminal Domain Inhibitors (BETis)

**Figure 5:**
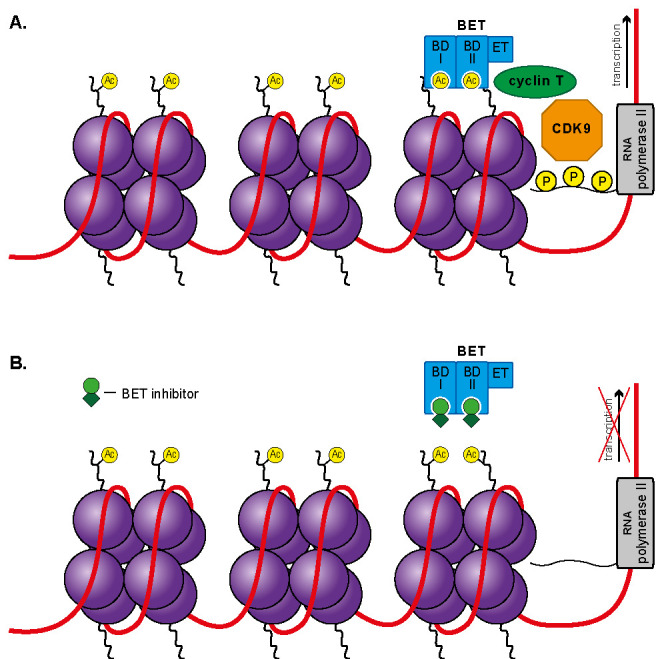
Visualization of the bromodomain and extra-terminal domain (BET) contribution in transcription initialization (**A**) and its suppression in the presence of BET inhibitors (**B**).

The bromodomain and extra-terminal domain (BET) family of proteins, also known as “Bromodomain,” consists of BRDT, BRD2, BRD3, and BRD4. All family members include two tandem bromodomains (BD I and II) and extra-terminal domain (ET) [131,132]. This structure determines the bromodomain’s ability to bind with recognized acetyl groups of histone tail lysines and their aptitude as chromatin readers. The other activity of BET proteins is recruiting the ET domain-specific transcription factors, such as transcription elongation factor b (Figure 5A) [133,134]. The BET family is responsible for stimulating the transcription of plenty of genes due to their ubiquitous occurrence in body tissues [134].

BETis are small molecules with an affinity for competitive inhibition. Thus, they impede the formation of the bromodomain–lysine acetyl group complex via the occlusion of bromodomain’s active site [132,135]. As such, both enlistments of transcription factors and ensuing gene expression are prevented (Figure 5B).

The excessive synthesis of proteins encoded by *Gli1* and *Gli2*, which are elements of the Hedgehog pathway, results in the development of SHH medulloblastoma overexpression of the downstream genes affecting the cell proliferation (*CCND1*, *CCND2*, *CCNE*, *MDM2*), stem cell regeneration (*GREM1*), cell survival (*BCL2*, *CFLAR*), and angiogenesis (*VEGF*) [136,137,138]. In physiological conditions, the transcription of *Gli* is suppressed by Suppressor of Fused (SUFU) as a result of the inhibition of the Smoothened protein by Protein Patched Homolog 1 (PTCH1). However, it may be stimulated directly regardless of other pathways [139]. For this reason, the invention of SMO inhibitors (like Vismodegib), which impair increased *Gli* transcription in the case of HH pathway deregulation, appears to be insufficient for MB therapy. Recent studies revealed I-BET151, which belongs to the group of BETis, as a likely drug for MB therapy [139]. Through BRD4, it hinders oncogenesis by diminishing *Gli* and downstream genes’ transcription, and *Smo* and *Ptch1* in *Sufu^−/−^* MEFs and SAG-activated Light2 cells. It has been assessed that I-BET151 has the most depletive influence on *Gli1* expression. Moreover, the administration of I-BET151 to *Ptch1^+/−^*-derived MB attenuates *Gli1*’s relative expression in a dose-dependent manner to about 20% [139]. Such promising test results lean toward more accurate testing I-BET151, with possible clinical introduction for the treatment of SHH medulloblastoma and other cancers with retained HH pathway-independent *Gli* activity.

The family of MYC genes contains three proto-oncogenes: *c-MYC* (*MYC*), *l-MYC* (*MYCL*), and *n-MYC* (*MYCN*). They encode proteins that deactivate cell-cycle inhibitors (p21, p27), increase proliferation via inducing cyclins activity (cyclin D, cyclin E, CDK2, CDK4), enhance stem cell renewal, apoptosis (Bcl-2 genes family inhibition), assurance of nutrients (GLUT1 and LDH synthesis), and angiogenesis stimulation (VEGF, IL-1β) [140,141]. In *MYC*-driven medulloblastoma, the amplification or overexpression can concern the entire family or only *c-MYC* or *n-MYC* [142]. The mechanism of *MYC* transcription is similar to *Gli* and relies on bromodomains, especially BRD4 acting as an expression-activating agent [134].

*Thienotriazolodiazepine* (*JQ1*) is a small molecule that binds to BET proteins with a distinct potency [135]. The highest specificity has been detected toward BRD4. A significant effect has been noticed in cell lines with elevated expression of BRD4 (HD-MB3, ONS-76, UW-228, D425, D458, D341, D283) compared with Daoy, which expresses BRD4 to a lesser extent. The least vulnerability on JQ1 has appeared in the Daoy and UW-228 lines. In contrast, HD-MB3 sensitivity has reached approximately 80% [134]. Moreover, after JQ1 application in cell colonies, augmentation of the G₁ fraction and diminished capability for proliferation were assessed [134,143]. It was induced using the depleted expression of *CCND1* and inactivation of E2F1 via maintaining RB phosphorylation using p21 and p27 [144]. Moreover, *MYC*-escalated transcription indicates stem cell self-renewal, which correlates with the insufficient synthesis of neural stem cell differentiation markers, such as MAP2. The treatment with JQ1 exhibited intensification of MAP2 expression and also reduced the level of stem cell markers, such as Nestin, Nanog, and SOX2. The distinctive therapy corollaries pertain to medulloblastoma cells, which harbor *TP53* mutation. In comparison with wild-type *TP53* cells, mutant cells manifest an impaired response to JQ1 [134]. Therefore, JQ1 occludes *MYC* transcription, supports MB cell apoptosis and senescence, disallows proliferation and, consequently, self-renewal. Nonetheless, its remedial impact may be abolished in certain cases. According to quoted findings, it can be assumed that JQ1’s multidimensional activity will have a crucial meaning in the treatment of *MYC*-driven MB, mostly occurring in subgroups WNT and group 3.

### 3.5. lncRNAs

MB group 3 has a markedly unfavorable prognosis. However, recent studies showed a connection between the MB subgroup and long noncoding RNAs. It has been shown that *lnc-HLX-2-7* is markedly overexpressed in this entity compared with other medulloblastoma subgroups [79]. This is a consequence of increased *MYC* transcription, which is frequently observed in group 3. *MYC* is an upstream regulatory gene for *lnc-HLX-2-7*. To assess the direct impact of *lnc-HLX-2-7* on tumor growth, its expression was suppressed in MED211 and D425 Med cell lines. The observed effects were elevated apoptosis and a decline in cell viability, proliferation, and colony formation ability. Moreover, the intensity of metabolism reactions, such as sirtuin pathways, oxidative phosphorylation, and mitochondrial reactions, changed [79]. Similar results were achieved with the JQ1 application, which inhibits *lnc-HLX-2-7* transcription via MYC synthesis obstruction. This suggests the possibility for the intervention with *lnc-HLX-2-7* inhibitors, which may facilitate overcoming the emerging resistance to BET inhibitors.

### 3.6. LSD1 and EZH2 Inhibitors

Lysine-specific histone demethylase 1A (LSD1; KDM1A) leads to the hypomethylation of H3K4,9 [145]. Cancer cells show higher expression of LSD1 than normal ones, and its overexpression results in a poor prognosis for the patients [33]. LSD1 expression is prominently increased among WNT, SHH, and group 3 compared to group 4 medulloblastoma [146].

KDM1A knockdown leads to apoptosis and blocks the proliferation and migration of MB cell lines. One of the first specific KDM1A inhibitors tested in MB cells was *NCL-1*. This small molecule significantly suppresses the cellular growth in Daoy and ONS-76 MB cell lines [33]. *SP2509* is a selective inhibitor of LSD1 that also prevents the development of MB cells. It was demonstrated that Daoy, ONS-76, and D283med MB cell lines are not able to grow because of LSD1 inhibition [147].

GFI1 and GFI1B were identified as MB oncogenes that are recurrently activated in groups 3 and 4 [148]. The interaction between LSD1 and growth factor independent 1 (GFI1) was shown in GFI1-driven medulloblastoma. Among the GFI1 family proteins, the SNAG (SNAIL/GFI1) domain was highlighted as being crucial for GFI1-driven tumorigenesis [149]. SNAG is an N-terminal transcriptional repression domain that is responsible for the recruitment of LSD1 to GFI1-regulated genes [150]. *T-3775440* is an irreversible LSD1 inhibitor that disrupts the interaction between LSD1 and SNAG domain protein GFI1B. The ability of this novel inhibitor to block cell line growth was demonstrated in a subset of human acute myeloid leukemia [151].

The other LSD1 inhibitor, *ORY-1001*, also effectively inhibited the growth of GFI1-driven tumors, suggesting that the suppression of LSD1 interactions is a strategy that is worth further consideration [149].

Patients with SHH medulloblastoma show increased LSD1 and REST expression, which entails hypoxia-inducible factor 1-alpha (HIF1A) expression [146]. HIF1A is a transcriptional regulator that, e.g., induces the transcription of genes involved in cell proliferation or migration. HIF1A was observed in many tumors, including gliomas. LSD1 inhibition results in decreased HIF1A expression. This process could be used as a therapeutic solution for patients with SHH medulloblastoma [146].

Some of the LSD1 inhibitors that are currently in clinical trials for cancer therapy are CC-90011, GSK-2879552, IMG-7289, INCB059872, ORY-1001, ORY-2001, and TCP. These inhibitors are mainly tested as potential therapies for small lung cancer cells and acute myeloid leukemia [145].

Enhancer of Zeste 2 (EZH2) is a histone-lysine N-methyltransferase that constitutes a part of the Polycomb Repressive Complex 2 (PRC2). PRC2 is required for long-term epigenetic silencing of chromatin and is responsible for regulating genes that are connected with embryonic development [152]. This complex catalyzes H3K27 methylation. PRC2 mutations and misregulations are associated with the development of various types of cancer, including medulloblastoma [153]. Significantly increased PRC2 expression is linked with poor prognosis. It was observed that mutations in EZH2 cause upregulation in PRC2 [154]. DZNep, EI1, EPZ005687, GSK343, GSK126, UNC1999, EPZ-6438, and the stabilized a-helix of EZH2 peptide (SAH-EZH2) are potential EZH2 inhibitors in clinical development. This proves that an EZH2 inhibitor is a potential therapeutic option [155].

### 3.7. Inhibition of MBD2

Recent studies revealed that the adhesion G-protein-coupled receptor B1 (*ADGRB1*) gene undergoes epigenetic silencing in medulloblastoma. *ADGRB1* encodes brain-specific angiogenesis inhibitor 1 (*BAI1*), which shows a p53-binding site [156]. *BAI1* prevents p53 Mdm2-mediated degradation. All medulloblastoma subtypes exhibit a lack of *BAI1* expression, resulting in the possibility of faster tumor development. Methyl-CpG Binding Domain Protein 2 (MBD2) plays a significant role in *ADGRB1* gene silencing. MBD2 pathway inhibition can reactivate *ADGRB1* expression following *BAI1* and p53 expression. Therefore, an MBD2 pathway inhibitor could be considered as a potential therapeutic target in patients with medulloblastoma [156].

### 3.8. Combined Therapies

Tumors are genetically diverse and consist of different populations of cells. Single-drug therapies cannot be effective enough because some cells can be resistant to that treatment, whereas others will respond well. Therefore, combination therapies are the necessary solution [157].

More effective treatment with DAC is achieved by combining it with other drugs, such as HDACs (e.g., VPA) [126]. This combination was studied on PTCH-related tumors, but despite inhibiting tumor formation, it was less effective in slowing down tumor growth in advanced stages, which may be independent of PTCH signaling [158]. The synergistic effect was also observed between DAC and SAHA, as well as DAC and parthenolide. Daoy and D283med cells exposed to these combinations underwent apoptosis and widespread cell death, and the decrease in cell viability was related to the concentration of the substances [110].

Another potential therapeutic combination is the following triple combination: DAC, abacavir, and irradiation. It was tested in three human MB cell lines: Daoy, MEB-Med8a, D283med. Abacavir is a carbocyclic nucleoside reverse transcriptase inhibitor and has potential in cancer treatment through inhibiting telomerase activity. It has the ability to reduce the proliferation of medulloblastoma cells. Moreover, abacavir that is used in combination with DAC results in a more substantial reduction in the number of all tested cells. Irradiation also acts in a synergistic manner, suggesting a new therapeutic option. It is worth mentioning that the triple combination does not show any toxicity on neural progenitor cells [157]. The other triple combination tested on medulloblastoma cells consisted of DAC, pan-HDAC inhibitor 4-phenylbutyrate, and a tyrosine kinase inhibitor, namely, imatinib. This combination triggered apoptosis in Daoy and UW228 in group 3 medulloblastoma cells [159].

The CDK inhibitors can occur as well-matched enhancers for JQ1 in view of CDKs’ necessary presence during the cell cycle. To assess the utility of a combined treatment, the activity of three CDK inhibitors, namely, Purvalanol A (acting on CDK1, CDK2, and CDK4), Milciclib (selective CDK2 inhibitor), and Palbociclib (selective CDK4/6 inhibitor), was examined on human group 3 MB *MYC*-overexpressing lines (D283, MB002, and sD425) and *MYCN*-amplified GTML2 cells [160]. Furthermore, Purvalanol’s higher efficacy toward neural stem cells (NSCs) and MB cells led to the annihilation of about 75% of valid NSCs as compared to the combination of JQ1 with Milciclib or Palbociclib, which reduced the amount of extinct NSCs to 30 and 42%, respectively [160]. Furthermore, the combination of JQ1 with Milciclib exhibits higher efficiency and rapidity in inducing apoptosis and cell-cycle arrest in both *MYC* and *MYCN*-driven cell lines in comparison with the combination of JQ1 with Palbociclib. It is worth mentioning that either Milciclib or Palbociclib alone and combined with JQ1 show insignificant impact on the MB non-*MYC*-amplified line Daoy. Another relevant example of a CDK inhibitor cooperating with JQ1 is THZ1 (CDK7 inhibitor). This association caused depleted *Gli1* and *Gli2* transcription and proliferation, together with raised apoptotic disintegration, both in SMO-inhibitor-sensitive (SMB21) and -resistant (SMB21-shSufu, SmoD477G-MB, A673, ATRT-03) cell lines [161]. Similar observations were noticed during an evaluation of birabresib (BET inhibitor MK-8628) and CT7001’s (CDK7 inhibitor) cumulative effect on SmoD477G-MB, SmoWT-MB, A673, and ATRT-03 cells [161]. Because of their influence on *Gli* expression and the survivability drop, they entered early-phase clinical trials. Synchronic use of BET and CDK7 inhibitors eradicate HH-derived medulloblastoma. However, it adversely alters healthy cells, which will demand restrictive treatment logistics in future attempts.

The aforementioned small molecule MK-8628 acts similarly to JQ1 by inhibiting BRD4-dependent *MYC* expression [162]. Polo-like kinase 1 inhibitors are enzyme PLK1 antagonists. They lead to cell cycle arrest through canceling phosphorylated MYC protein stability via occluding its binding to PLK1 [163]. Both compounds have similar properties: they decrease proliferation and self-renewal, diminish cell viability, induce apoptosis, and exempt tumor expansion in HD-MB03, Daoy, UW228, and ONS-76 cell lines. Despite this anti-medulloblastoma activity, neither the MK-8628 nor PLK1 inhibitor alone has shown curative effects. However, in two combined therapies (BRD4 inhibitor and PLK1 inhibitor), namely, MK-8628 with Volasertib and MK-8628 with GSK461364A, greater redundancy of MYC downstream genes’ (i.a. *CCND1* and *WEE1*) expression was detected in comparison to a single treatment [162]. Even though the combined therapy seems to be more effective against *MYC*-driven MB, it presents greater unpredictability regarding pharmacokinetic reactions. Consequently, the administration of two drugs results in difficulties in treatment planning and the accumulation of side effects. For this reason, a recent discovery of dual BRD4/PLK1 inhibitors has appeared to be an attractive alternative. Among these compounds, UMB103 and UMB160 are characterized with selective antitumor activity concomitantly with a minor impact on properly functioning cells [163]. It was evinced via UMB103 and UMB160 application on *MYC*-amplified HD-MB03 line cells, which has contributed to reduced *MYC* and *MYCN* expression, proliferation, and cell viability simultaneously with enhanced apoptosis [163]. The current data suggest that therapy with BETis and BRD4/PLK1 inhibitors has high efficacy and great potential for further development in favor of patients’ better recovery. Table 6 summarizes the selected currently known combined therapies.

## 4. Conclusions

Our article shows the perspectives and benefits of using epigenetic mechanisms in the treatment of MB. Since each described mechanism is based on the tumor development pathway, it allows for creating targeted and, therefore, more effective therapies. It also emphasizes that knowledge of the epigenetic basis of carcinogenesis is crucial for developing and choosing an adequate tumor treatment. Moreover, such therapies can act selectively on cancerous cells, which is associated with less damage and side effects to healthy tissue. However, it is significant that drug combinations of epigenetic agents, also with currently used chemotherapeutics, present enhanced efficiency in comparison to monotherapies. Numerous potential benefits, such as less toxicity and better targeting, confirm that epigenetic mechanisms are a promising direction for developing novel therapies against medulloblastoma.

## Figures and Tables

**Table 1:** Clinical, histological, and molecular characteristics of medulloblastoma subtypes [7,15,19,20,21,22,23].

Feature	WNT	SHH	Group 3	Group 4
Occurrence	10%	30%	25%	35%
Age group	Older children	<3 y.o. and >16 y.o.	Infants, young children, and adults	Children of all ages and adults
Male:female ratio	1:1	1:1	2:1	3:1
Location	Cerebellar peduncle, cerebellopontine angle cistern	Cerebellar hemispheres, midline vermis, fourth ventricle	Midline vermis, fourth ventricle	Midline vermis, fourth ventricle
Metastasis	Rare (5–10%)	Uncommon (15–20%)	Very frequent (40–45%)	Frequent (35–40%)
Prognosis	Low-risk	Low-risk in infants, standard risk in others	High-risk tumor	Standard risk
Survival	>90%	75%	50%	75%
Histological subgroup	Classic LC/A	Classic LC/A DN MBEN	Classic LC/A	Classic LC/A
Carcinogenesis pathway	WNT	SHH	Photoreceptor/ GABA	Neuronal/ glutamatergic
Single nucleotide variants	*CTNNB1* *DDX3X* *SMARCA4* *TP53*	*SMO* *PTCH1* *SUFU* *TERT* *TP53*	*SMARCA4* *KBTBD4* *KMT2D* *CTDNEP1*	*KDM6A* *KTM2C* *ZMYM3* *KBTBD4*
Gene amplification	NA	*GLI1* *GLI2* *MYCN*	*OTX2* *MYC* *MYCN*	*CDK6* *OTX2* *MYCN* *SNCAIP*

**Table 2:** Dysregulated miRNAs in MB and their functional targets.

miRNA	Upregulation (↑) or Downregulation (↓)	Functional Target	Ref.
miR-17/92	↑	SHH pathway, MYCN/MYC, Gli 1	[49]
Let-7g		RAS, STAT3	[50]
miR-199-5p		HES 1, Notch pathway, ErbB2	[51]
miR-214		SHH pathway, Gli 1	[52]
miR-100		BTG2	[53]
miR-106b		-	[53]
miR-9	↓	REST/NRSF, t-Trk-C	[52]
miR-125a			[54]
miR-124a		CDK6	[55]
miR-125b		Smo, Gli 1	[56]
miR-324-5p			[57]
miR-326			[45]
miR-218		EGFR, Bcl-2	[58]
miR-584-5p		eIF4E3v, HDAC1	[59]
miR-34a		TP53	[60]
miR-128a		BMI1	[61]

**Table 4:** Summary of presented epigenetic mechanisms dysregulations in medulloblastoma.

Epigenetic Mechanism	Enzyme/ Molecule	Type of Dysregulation	Action	Effect in MB
Histone methylation	Histone demethylase KDM1A	Overexpression	Hypo- methylation of H3K4	Poor prognosis
Histone Acetylation	Deacetylases	-	Silences *DKK1*	Promotes the WNT signaling pathway
miRNA	miR-326	Downregulation	Promotes mRNA translation	Promotes the SHH signaling pathway
DNA methylation	Methyl- transferases	Overexpression	Hyper- methylation of anti-oncogenes promotors	Silences anti-oncogenes

**Table 5:** Selection of the most researched histone deacetylase inhibitors in medulloblastoma.

Histone Deacetylase Inhibitors	Chemical Structure	Target Histone Deacetylases (HDACs)	Function	Combined Treatment	Ref.
Suberoylanilide hydroxamic acid (SAHA, Vorinostat)	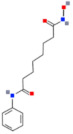	Classes I and II	Activation of the *p21^WAF1^*, decline of REST, REST-dependent repression of cell growth	Chemotherapeutics (Doxorubicin, Etoposide, and Cisplatin), → caspase-dependent apoptosis	[109,110,111,112]
Panobinostat (LBH-589)	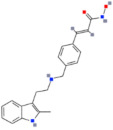	Pan-HDACi	Increase of the *FOXO1* target genes, down-regulation of *ID3*	PI3Ks inhibitors, → inhibition of growth of *MYC*-driven MB	[113,114]
Trichostatin A (TSA)	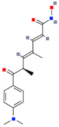	Classes I and II	Upregulation of *DKK1*, acetylation of *Gli1* and *Gli2*	Cul3–REN E3 ubiquitin ligase complex, → suppression of MB growth	[38,115,116]
Valproic acid (VPA)	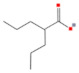	Classes I and II	Activation of *p21*; suppression of *TP53*, *CDK4*, and *c-MYC* expression	DAC → prevention of Ptch-associated tumor formation	[117,118]
Sodium butyrate (NaB)	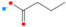	Classes I and IIa	Downregulation of *CD133*, *BMI1*, and ERK activity	(MAPK)/ERK inhibition, → antiproliferative effect	[113,114,115]
Quisinostat (JNJ-26481585)	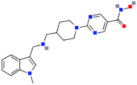	Pan-HDACi	Down-regulation of the SHH target genes, especially *Gli1*; induction of caspase-3 and PARP cleavage	-	[116,117,118]
Entinostat (MS-275)	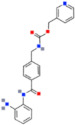	Class I	Acetylation of the Ku70 protein	Chemotherapeutics (Doxorubicin, Etoposide, and Cisplatin), → caspase-dependent apoptosis	[112]

**Table 6:** The list of the combined therapies that are available against medulloblastoma.

Drug Combinations	Results	Ref.
DAC + VPA	Reduction of tumor cell viability in Daoy and D283med lines	[97,110]
DAC + SAHA	Induction of apoptosis in Daoy and D283med cells	[110]
DAC + parthenolide		
DAC + abacavir + irradiation	Reduction of tumor cells in Daoy, MEB-Med8a, and D283med cell lines	[164]
DAC + 4-phenylbutyrate + imatinib	Induction of apoptosis in Daoy and UW228 in group 3 MB cells	[159]
JQ1 + Milciclib or Palbociclib	Induction of apoptosis and cell-cycle arrest in *MYC*, *MYCN*-derived, and Daoy cell lines	[160]
JQ1 + THZ1	Reduction of *Gli1* and *Gli2* transcription and proliferation with increased apoptosis in SMB21, SMB21-shSufu, SmoD477G-MB, A673, and ATRT-03 cell lines	[161]
MK-8628 + CT7001	Reduction of Gli expression and viability	[162]
MK-8628 + Volasertib or GSK461364A	Reduction of proliferation, tumor cell viability, and expansion, as well as the induction of apoptosis in HD-MB03, Daoy, Uw228, and ONS-76 cell lines	[163]
UMB103 or UMB160 *	Reduction of *MYC* and *MYCN* transcription, proliferation, and viability with increased apoptosis in HD-MB03 cells	[163]

* One drug with a dual mechanism of action.
